# The Fly Maggot Antioxidant Peptide (FMP) Alleviates Oxidative Damage in the Intestines of Weaned Piglets by Enhancing Mitochondrial Autophagy Through Activation of the Nrf2 Signaling Pathway

**DOI:** 10.3390/antiox15070791

**Published:** 2026-06-24

**Authors:** Xingke Wang, Ruiying Bao, Qingchao Yang, Qian Yang, Sheng Gao, Qingying Cai, Yang Zhang, Haiwen Zhang, Huiyu Shi, Xuemei Wang

**Affiliations:** Animal Nutrition and Feed Laboratory, School of Tropical Agriculture and Forestry, Hainan University, Danzhou 571737, China; xkwang28@hainanu.edu.cn (X.W.); 23110710000036@hainanu.edu.cn (R.B.); 24220951330003@hainanu.edu.cn (Q.Y.); 20233004953@hainanu.edu.cn (Q.Y.); 23210905000009@hainanu.edu.cn (S.G.); 23220951330001@hainanu.edu.cn (Q.C.); 25210905000015@hainanu.edu.cn (Y.Z.); hwzhang@hainanu.edu.cn (H.Z.); 993978@hainanu.edu.cn (H.S.)

**Keywords:** antioxidant peptide, oxidative stress, mitophagy, weaned piglets, intestinal barrier

## Abstract

Intestinal oxidative stress severely compromises the health and growth of weaned piglets. The fly maggot-derived antioxidant peptide FMP was previously identified, but its protective mechanisms remain unclear. Here, we explored how FMP alleviates oxidative intestinal injury. In IPEC-J2 cells, FMP pretreatment significantly attenuated H_2_O_2_-induced cytotoxicity, ROS accumulation, and apoptosis, while enhancing antioxidant enzyme activities and activating Nrf2 signaling (*p* < 0.05). Co-treatment with the Nrf2 inhibitor ML385 abolished FMP-mediated mitophagy enhancement and cytoprotection, revealing that FMP enhances PINK1/Parkin-dependent mitophagy via Nrf2 activation. In diquat-challenged weaned piglets, oral FMP administration restored serum SOD and GSH-Px activities, reduced MDA and DAO levels (*p* < 0.05), upregulated jejunal tight junction proteins, and enriched *Lactobacillus* populations. These findings demonstrate that FMP targets the Nrf2-mitophagy axis to protect against intestinal oxidative damage, supporting its application as a green feed additive.

## 1. Introduction

In intensive livestock production, oxidative stress represents a major bottleneck restricting both profitability and animal welfare [1]. Multiple stressors—dietary shifts, temperature and humidity extremes, high stocking density, and regrouping—persistently disrupt redox homeostasis, reducing feed intake, nutrient utilization and immune competence, and ultimately causing growth retardation and poor performance [2,3]. Weaned piglets are particularly susceptible owing to their immature gut and weak endogenous antioxidant capacity. Weaning-associated stressors (maternal separation, abrupt dietary change, environmental shifts) acutely exacerbate reactive oxygen species (ROS) accumulation, giving rise to anorexia, persistent diarrhea, intestinal mucosal atrophy, epithelial apoptosis, and, in severe cases, mortality rates of 15–20%, leading to heavy economic losses [4,5]. The intestine is a primary target of oxidative stress. Excess ROS compromise intestinal barrier integrity (physical, chemical, immunological and microbial components) by disrupting enterocyte tight junctions, inducing apoptosis, perturbing the gut microecology and attenuating digestive enzyme secretion, ultimately resulting in nutrient malabsorption and systemic inflammation—the core pathological basis of oxidative stress-mediated growth suppression in piglets [6,7].

Endogenous defense against oxidative stress revolves around two principal and synergistically interacting axes: mitochondrial homeostasis and the Keap1/Nrf2 pathway [8]. Mitochondria are the main intracellular source of ROS; electron leakage from the respiratory chain damages mtDNA and respiratory complexes, establishing a vicious cycle of ROS generation–mitochondrial injury–augmented ROS release [9,10]. Mitophagy, which selectively clears damaged mitochondria, breaks this cycle at its origin, preserves intestinal epithelial energy homeostasis and suppresses apoptosis, thereby safeguarding barrier integrity [11]. In the Keap1-Nrf2 system, Keap1 constitutively sequesters Nrf2 and promotes its proteasomal degradation. Upon oxidative challenge, redox-sensitive modifications of Keap1 release Nrf2, which accumulates, translocates to the nucleus, and activates antioxidant response element (ARE)-driven transcription of cytoprotective enzymes (e.g., SOD, CAT, GSH-Px, HO-1). This coordinated response scavenges surplus ROS and repairs oxidative lesions, restoring cellular redox balance [12,13]. The cooperative action of these two pathways constitutes the core cellular defense network and provides key targets for antioxidant intervention.

Given the limitations of endogenous defenses under sustained or severe oxidative challenge, exogenous antioxidants are essential. Conventional synthetic antioxidants (e.g., BHT, BHA) face stringent regulatory restrictions because of potential genotoxic and carcinogenic risks [14]. Antioxidant peptides (2–20 amino acids) derived from natural sources are safe, low-molecular-weight, and highly bioavailable, acting through direct radical scavenging, metal-ion chelation, and potentiation of endogenous antioxidant signaling [15,16]. Insect-derived proteins and peptides offer sustainable advantages [17]; notably, maggot-derived antioxidant peptides exhibit strong free-radical-scavenging capacity and gastrointestinal digestive stability and have been shown to alleviate oxidative stress injury and preserve intestinal barrier architecture in cellular models and livestock trials [18]. However, current research remains largely descriptive, and a systematic elucidation of their specific molecular targets, the core regulatory pathways they engage, and—critically—their mechanistic interplay with mitophagy and the Nrf2 pathway is lacking. This knowledge gap impedes their precise and large-scale application.

In previous work, we hydrolyzed maggot larvae and identified an antioxidant peptide with the sequence YDCFQQN, designated FMP (fly maggot antioxidant peptide). In the present study, we first use in vitro cell assays to characterize the principal signaling cascades through which FMP modulates oxidative injury and to clarify the coordinated regulation of Nrf2 pathway activation and mitophagy. Subsequently, we perform an in vivo investigation in weaned piglets to verify the capacity of FMP to mitigate systemic oxidative stress and improve intestinal health indices. The central aim is to systematically decipher the molecular determinants underlying the protective actions of maggot-derived antioxidant peptides against intestinal oxidative insult, thereby furnishing a sound theoretical basis for their industrial development and practical deployment in swine production.

## 2. Materials and Methods

### 2.1. Chemicals and Reagents

FMP (purity > 98%) was procured from Gen Script (Nanjing, China). The preliminary identification of the antioxidant peptide was carried out by extracting and purifying peptides from *Musca domestica* larvae, determining the peptide sequence via liquid chromatography-tandem mass spectrometry (LC-MS/MS), and verifying its antioxidant activity through solid-phase synthesis. NAC (N-Acetylcysteine) was obtained from Biosharp (Beijing, China). DQ (Diquat) and ML385 were obtained from Target Mol (Boston, MA, USA). Assay kits for the determination of total antioxidant capacity (T-AOC), SOD, GSH-Px, CAT, malondialdehyde (MDA), D-lactic acid, and diamine oxidase (DAO) were purchased from Jiancheng Bioengineering Institute (Nanjing, China). Enzyme-linked immunosorbent assay (ELISA) kits for the detection of interleukin-6 (IL-6), interleukin-10 (IL-10), interleukin-1β (IL-1β), and tumor necrosis factor-alpha (TNF-α) were obtained from Meibiao Biotechnology (Nanjing, China). The following items were sourced from Service Biotechnology Co., Ltd. (Wuhan, China): CCK-8 assay kit, hematoxylin and eosin (H&E) staining kit, RIPA lysis buffer, Dulbecco’s modified Eagle medium (DMEM), fetal bovine serum (FBS), penicillin–streptomycin (PS), phosphate-buffered saline (PBS), Alexa Fluor 488-conjugated secondary antibodies, Hoechst 33258 staining kit, SDS-PAGE gels, caspase-3 detection kit, and enhanced chemiluminescence (ECL) reagent kit. Primary antibodies recognizing ZO-1, Occludin, Claudin-1, VDAC, Parkin, PINK1, p62, and LC3 were sourced from Bioss Antibodies (Beijing, China). Wanlei Biotechnology Co., Ltd. (Shenyang, China) provided antibodies against p-MAP3K, p-JNK, cyt c, and Bax. Antibodies specific for Keap1, Nrf2, NQO1, HO-1, and β-actin were purchased from Santa Cruz Biotechnology (Dallas, TX, USA).

### 2.2. Cell Culture and Treatments

The IPEC-J2 porcine small intestinal epithelial cell line was obtained from Beina Biotechnology (Xinyang, China). Cells were maintained in Dulbecco’s modified Eagle medium (DMEM) containing 5% fetal bovine serum (FBS), 5% penicillin–streptomycin (PS), 1% insulin–transferrin–selenium (ITS), and 5 ng/mL epidermal growth factor (EGF). Incubation was performed at 37°C within a humidified atmosphere of 5% CO_2_. For all treatment experiments, IPEC-J2 cells were seeded in 6-well plates at a density of 5 × 10^5^ cells per well and cultured for 24 h before treatment. Cells were then pre-incubated for 1 h with either PBS alone or ML385 at a final concentration of 5 μM (equivalent to 20 nmol/10^6^ cells, based on a 2 mL culture volume). Thereafter, cells were exposed to various concentrations of FMP (e.g., 50–200 μg/mL) for 24 h, followed by challenge with H_2_O_2_ at 1000 μM (equivalent to 4 μmol/10^6^ cells) for an additional 4 h. After treatment, cells were rinsed twice with ice-cold PBS and collected for subsequent analyses.

### 2.3. Experimental Animals and Treatments

All animal procedures were approved by the Animal Ethics Committee of Hainan University (Approval No. IAS2025-00206). A total of 24 weaned Wuzhishan piglets (35 days of age; body weight 3.12 ± 0.28 kg; equal distribution of males and females) were randomly allocated into four experimental groups: (1) control group (Con), (2) DQ group (Mod), (3) low-dose FMP plus DQ group (LFMP), and (4) high-dose FMP plus DQ group (HFMP). All animals included in this study had no prior experimental interventions. Animals that died unexpectedly during the experiment, developed severe surgical complications, or failed to complete the experimental procedures were excluded from the study. For data analysis, outliers defined as values exceeding three standard deviations from the group mean were excluded. All inclusion/exclusion criteria for animals and data points were pre-specified in the experimental protocol prior to the initiation of the study. A basal diet formulated to satisfy the nutritional requirements of weaned piglets was provided to all experimental groups. Piglets in the low-dose FMP (LFMP, 100 mg/kg) and high-dose FMP (HFMP, 200 mg/kg) groups were administered FMP dissolved in sterile physiological saline via daily oral gavage, while animals in the respective control groups received an equivalent volume of the saline vehicle alone through the identical route. At predetermined time points, piglets within the Mod, LFMP and HFMP groups were subjected to intraperitoneal injection of DQ (8 mg/kg body weight); simultaneously, those in the Con groups were given an intraperitoneal injection of an equal volume of sterile physiological saline. Throughout the entire duration of the experiment, all animals (pigs) were housed at a density of two pigs per pen under hygienic and comfortable environmental conditions. All pigs were provided with unrestricted access to drinking water ad libitum. At the conclusion of the trial (day 42), piglets were anesthetized and subsequently exsanguinated by cervical venesection, after which specimens of jejunal tissue, mucosal scrapings, and luminal digesta were harvested.

### 2.4. Histological Analysis

Morphological assessment of jejunal specimens fixed with 4% paraformaldehyde was conducted using a H&E staining kit. Tissue sections were sequentially subjected to dehydration, embedding, sectioning, and staining prior to examination under a Leica light microscope. For ultrastructural examination, jejunal tissue specimens and IPEC-J2 cells were immersed in 2.5% glutaraldehyde fixative and thereafter prepared for TEM. Ultrathin sectioning was performed by Wuhan Servicebio Technology Co., Ltd. (Wuhan, China).

### 2.5. Cell Viability, Redox Status, and Inflammatory/Barrier Marker Assays

Cell viability was determined using the CCK-8 assay. In brief, IPEC-J2 cells were seeded into 96-well plates at a density of 1 × 10^4^ cells per well and cultured for 24 h to permit attachment prior to the application of specified treatments for the requisite durations. After the treatment period, the culture supernatant was removed and substituted with fresh medium supplemented with 10 μL of CCK-8 solution per well, followed by a further 3-h incubation. The absorbance at 450 nm was then measured with a microplate reader. Antioxidant parameters in serum, jejunal mucosal homogenates, and IPEC-J2 cell lysates—specifically T-AOC, SOD, GSH-Px, CAT, and MDA content—were quantified using commercial biochemical assay kits according to the protocols provided by the manufacturers. Corresponding assay kits were likewise employed to determine serum concentrations of inflammatory mediators, namely IL-6, IL-10, IL-1β, and TNF-α, as well as markers of intestinal barrier integrity, including D-lactic acid and DAO.

### 2.6. 16S rRNA Amplicon Sequencing of the Microbiome

Jejunal digesta samples were subjected to total genomic DNA extraction with the MagPure Soil DNA LQ Kit (Magan, Guangzhou, Guangdong, China) according to the protocol provided by the manufacturer. The purified DNA served as the template for bacterial 16S rRNA gene amplification, which was performed using barcode-indexed primers together with Takara Ex Taq DNA polymerase (Takara). To profile the bacterial community, the V3–V4 hypervariable segment of the 16S rRNA gene was targeted via PCR employing the universal forward primer 343F (5′-TACGRAGGCAGCAG-3′) and reverse primer 798R (5′-AGGGTATCTAATCCT-3′). Library sequencing was carried out on the Illumina NovaSeq 6000 instrument with 250 bp paired-end chemistry, and the ensuing bioinformatic processing was handled by OE Biotech Co., Ltd. (Shanghai, China). Calculations of alpha and beta diversity indices were performed within the QIIME2 software (version 2025.10) environment. Beta diversity patterns were visualized via unweighted UniFrac principal coordinate analysis (PCoA). Intergroup differences were assessed for statistical significance using the Kruskal–Wallis test. Linear discriminant analysis effect size (LEfSe) was further applied to discern bacterial taxa with differential abundance among the experimental groups, employing a linear discriminant analysis (LDA) score threshold of 4.0 for biomarker identification.

### 2.7. ROS Measurement

Following exposure to the designated experimental conditions or the ROS-inducing positive control (ROSUP), the overlying medium was discarded and replaced with serum-free medium containing 10 μM of the ROS-responsive fluorogenic probe 2′,7′-dichlorodihydrofluorescein diacetate (DCFH-DA). Cells were then maintained for a 20-min interval at 37 °C within a humidified incubator. At the end of this incubation period, three successive rinses were performed using serum-free medium. Visualization of fluorescence signals was subsequently achieved with a laser scanning confocal microscope (ZEISS LSM800, Carl Zeiss Microscopy GmbH, Jena, Germany).

### 2.8. Immunofluorescence

Jejunal mucosal specimens were harvested from piglets immediately after euthanasia. Cells were subjected to three washes with PBS and then immersed in 4% paraformaldehyde for a 15-min fixation period. An additional triple-rinse sequence with PBS was performed prior to membrane permeabilization, which was achieved using Tris-buffered saline containing Tween 20 (TBST) for 30 min. Blocking of non-specific binding sites was carried out with 10% goat serum for 30 min. Subsequently, the preparations were exposed overnight at 4 °C to primary antibodies recognizing Claudin-1 (1:100 dilution), Occludin (1:100), and ZO-1 (1:100). Detection was then accomplished by applying Alexa Fluor 488-conjugated secondary antibodies (diluted 1:150) for 1 h at 37 °C in the absence of light. After three further PBS washes, nuclei were counterstained with 4′,6-diamidino-2-phenylindole (DAPI). Imaging was performed using an inverted fluorescence microscope (Leica DMi3000B, Leica Microsystems, Wetzlar, Germany).

### 2.9. Western Blot Analysis

Jejunal mucosal tissue and IPEC-J2 cell lysates were processed for total protein extraction using RIPA lysis buffer. The resulting protein preparations were fractionated by SDS-PAGE and subsequently transferred onto 0.45 μm polyvinylidene difluoride (PVDF) sheets (Millipore, Burlington, MA, USA). Blocking of the membranes was conducted for 2 h with a solution of 5% non-fat dry milk, after which they were exposed overnight at 4 °C to primary antibodies targeting the following molecules: ZO-1, Occludin, Claudin-1, β-actin, VDAC, Keap1, Nrf2, HO-1, NQO1, p-MAP3K, p-JNK, cyt C, Bax, PINK1, Parkin, p62, and LC3. The next day, membranes were probed with the appropriate secondary antibodies for a 1-h interval at ambient temperature. Immunoreactive signals were developed using an enhanced chemiluminescence (ECL) substrate kit, and densitometric analysis of the resultant bands was carried out with ImageJ software (version 1.64r).

### 2.10. Statistical Analysis

Results are presented as the mean value accompanied by the standard error (SEM). All statistical analyses were performed using R software (version 4.2.1). For pairwise evaluations, the significance of differences between two independent cohorts was determined using Student’s *t* test. In the case of comparisons encompassing three or more groups, statistical assessment was conducted via one-way analysis of variance (ANOVA), with subsequent pairwise contrasts examined using Tukey’s multiple comparisons procedure. The animal experiment followed a completely randomized design, with an equal number of males and females allocated to each treatment group to balance sex as a biological factor. Across all analyses, a calculated probability below the 0.05 threshold (*p* < 0.05) was taken to denote statistical significance.

## 3. Results

### 3.1. FMP Alleviates H_2_O_2_-Induced Oxidative Injury in IPEC-J2 Cells via Activation of the Nrf2 Signaling Pathway

**Figure 1:**
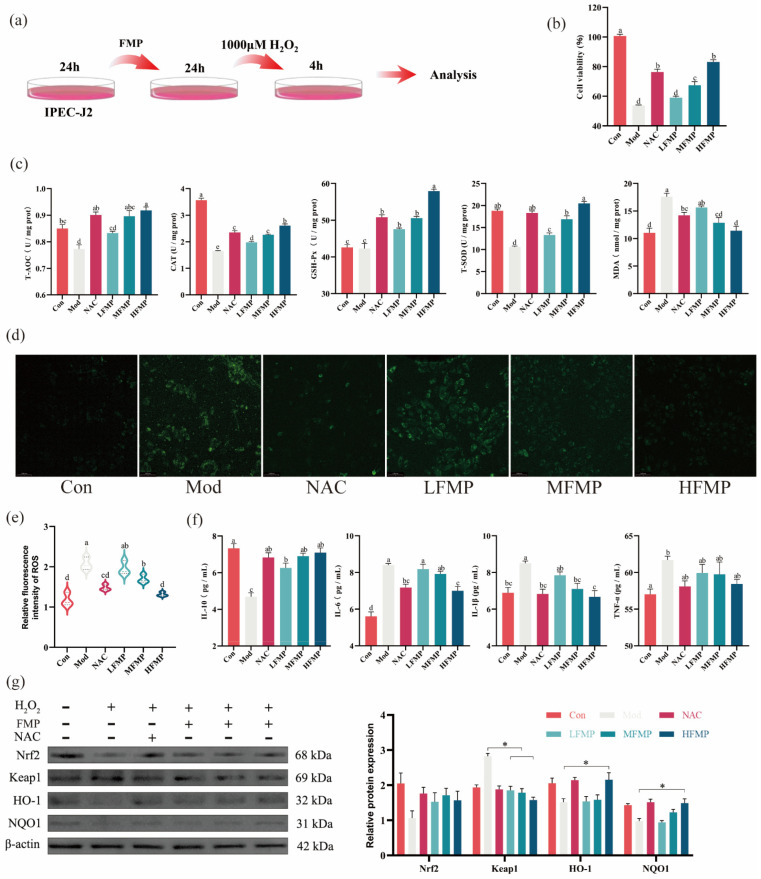
FMP increased antioxidant capacity in intestinal epithelial cells (IPEC-J2). (**a**) Schematic diagram illustrates drug administration in IPEC-J2 cells. (**b**) The cell viability of IPEC-J2 cells was determined by CCK-8 assay, *n* = 6. (**c**) The levels of T-AOC, CAT, GSH-Px, SOD, and MDA were determined by biochemical assay kits, *n* = 6. (**d**) Staining of cellular ROS was determined by ROS Assay Kit. (**e**) Relative content of cellular ROS, *n* = 3. (**f**) The levels of IL-10, IL-6, IL-1β, and TNF-α were determined by biochemical assay kits, *n* = 6. (**g**) Western blotting determined the protein expression and quantitation of Nrf2, Keap1, H0-1, and NQO1, *n* = 3. Values are means ± SEM. Different letters and * represent significant differences (*p* < 0.05).

To investigate the protective mechanism of FMP against H_2_O_2_-induced oxidative injury in IPEC-J2 cells, an oxidative stress model was established. Treatment with 1000 μmol/L H_2_O_2_ for 4 h reduced cell viability to approximately 50–60%, meeting criteria for an ideal injury model (Figure S1). Pre-incubation with 100, 250, and 500 μmol/L FMP for 24 h significantly improved viability, with higher concentrations exhibiting a declining trend in protective efficacy (Figure S2). Based on these results, for the subsequent cell experiments, IPEC-J2 cells were pretreated with FMP at concentrations of 100, 250, and 500 μM, followed by exposure to 1000 μM H_2_O_2_ to establish the oxidative injury model. The experimental groups were defined as Con (control), Mod (model, H_2_O_2_ alone), NAC (positive control, 500 μM NAC), LFMP (100 μM FMP pretreatment), MFMP (250 μM FMP pretreatment), and HFMP (500 μM FMP pretreatment) (Figure 1a), and the cytoprotective effect was further confirmed (Figure 1b). NAC, a widely used reactive oxygen species scavenger and glutathione precursor, served as the positive antioxidant control. H_2_O_2_-induced alterations in antioxidant enzyme levels were significantly ameliorated in the HFMP group (*p* < 0.05); notably, HFMP yielded significantly greater improvements in CAT, GSH-Px, and MDA levels compared with equivalent-concentration NAC (500 μM) (*p* < 0.05; Figure 1c). MFMP and HFMP pretreatment substantially suppressed intracellular ROS accumulation (Figure 1d,e). FMP upregulated IL-10 at different levels (*p* < 0.05), and, in the HFMP group, downregulated IL-6 and IL-1β (*p* < 0.05; Figure 1f). Western blot analysis revealed that H_2_O_2_ challenge decreased Nrf2, HO-1, and NQO1 protein levels while increasing Keap1; HO-1 and NQO1 levels were effectively reversed by HFMP and Keap1 levels were significantly improved at different concentrations of FMP (Figure 1g). Collectively, these results indicate that FMP activates the Nrf2/Keap1 antioxidant signaling axis to modulate antioxidant enzymes and inflammatory mediators, thereby mitigating oxidative insult in IPEC-J2 cells.

### 3.2. FMP Attenuates H_2_O_2_-Induced Oxidative Injury via Enhancement of Mitophagy and Suppression of Apoptosis

**Figure 2:**
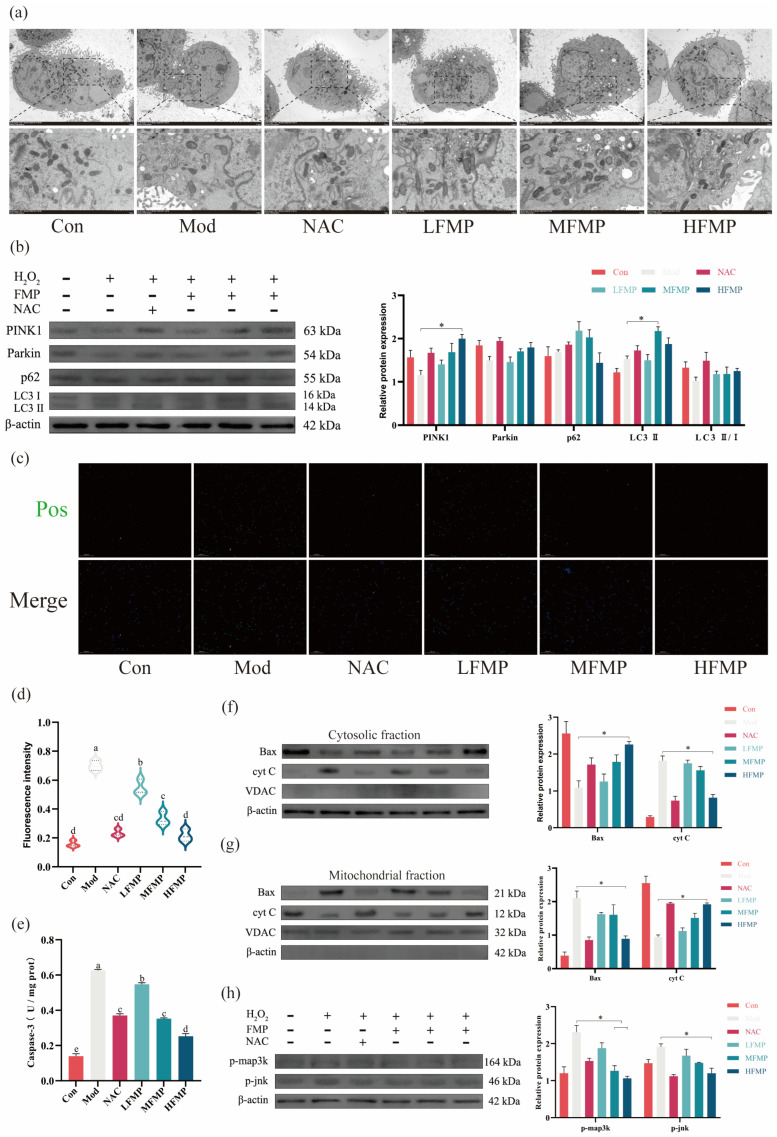
FMP attenuates H_2_O_2_-induced oxidative injury in IPEC-J2 cells via enhancement of mitophagy. (**a**) Cellular ultrastructure was visualized using TEM (2000 × magnification). (**b**) Western blotting determined the protein expression and quantitation of PINK1, Parkin, p62, LC3 I and LC3 II, *n* = 3. (**c**) Staining of apoptotic cells was determined by Tunel Assay Kit. (**d**) Relative content of apoptotic cells, *n* = 3. (**e**) The content of Cysteine-aspartic protease-3 was determined by Caspase-3 Assay Kit, *n* = 6. (**f**) Western blotting determined the protein expression and quantification of Bax and cyt C in the cytoplasm, *n* = 3. (**g**) Western blotting determined the protein expression and quantification of Bax and cyt C in the mitochondria, *n* = 3. (**h**) Western blotting determined the protein expression and quantification of p-MAP3K and p-JNK, *n* = 3. Values are means ± SEM. Different letters and * represent significant differences (*p* < 0.05).

FMP treatment potentiated mitophagy in H_2_O_2_-stressed IPEC-J2 cells. TEM revealed that H_2_O_2_ caused severe mitochondrial damage, characterized by vacuolization, cristae disruption and fragmentation, whereas FMP substantially preserved mitochondrial ultrastructure (Figure 2a). Concordantly, HFMP significantly increased PINK1, while MFMP significantly increased LC3-II (*p* < 0.05; Figure 2b). TUNEL staining showed a marked elevation of apoptotic cells in the model group, an effect significantly reversed by FMP (*p* < 0.05; Figure 2c,d). Mechanistically, mitochondrial dysfunction triggered cytochrome c release into the cytosol and Bax translocation to mitochondria in the model group, both of which were notably attenuated by FMP (Figure 2f,g). Moreover, the phosphorylation of MAP3K and JNK, key apoptotic regulators, was significantly enhanced in the model group and suppressed by HFMP (*p* < 0.05; Figure 2h). Caspase-3 cleavage exhibited a parallel trend (Figure 2e). Collectively, these results demonstrate that mitophagy is an integral component of the protective machinery through which FMP preserves mitochondrial integrity and restrains the mitochondrial apoptotic cascade in oxidatively injured intestinal epithelial cells.

### 3.3. FMP Attenuates H_2_O_2_-Induced Oxidative Injury in IPEC-J2 Cells via Nrf2-Dependent Promotion of Mitophagy and Suppression of Apoptosis

**Figure 3:**
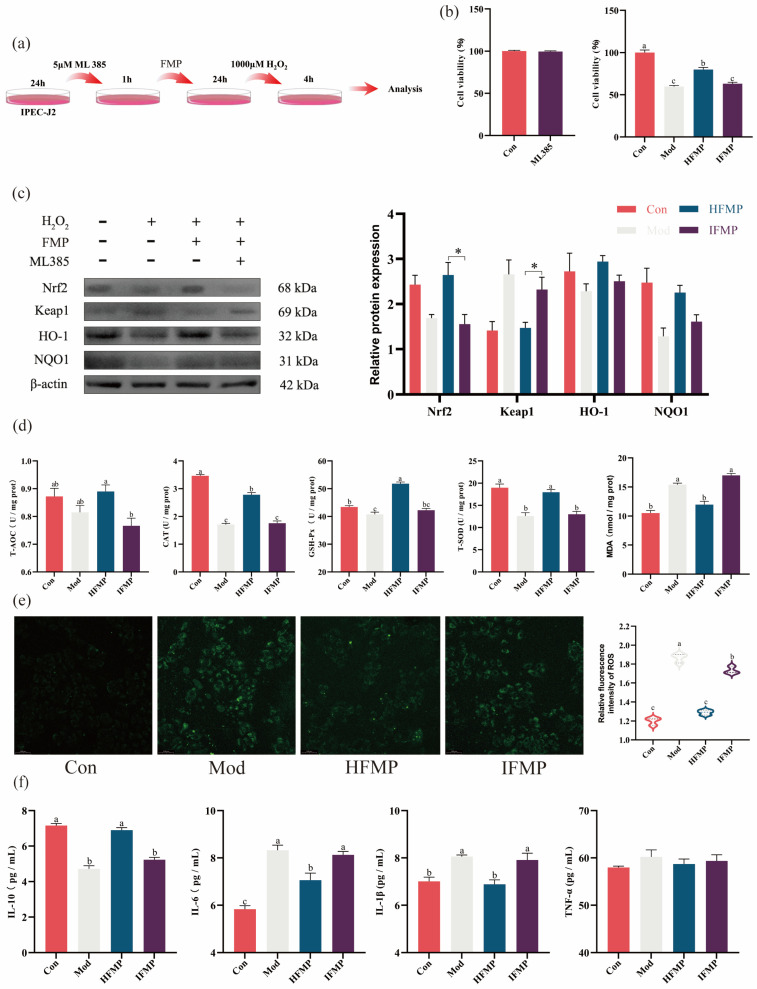
Nrf2 pathway inhibitor ML385 reverses the enhancement of antioxidant capacity conferred by FMP in IPEC-J2 cells. (**a**) The schematic diagram illustrates drug administration in IPEC-J2 cells. ML385, Nrf2 pathway inhibitor. (**b**) The cell viability of IPEC-J2 cells was determined by CCK-8 assay, *n* = 6. (**c**) Western blotting determined the protein expression and quantitation of Nrf2, Keap1, H0-1, and NQO1, *n* = 3. (**d**) The levels of T-AOC, CAT, GSH-Px, SOD, and MDA were determined by biochemical assay kits, *n* = 6. (**e**) Staining of cellular ROS was determined by ROS Assay Kit and relative content of cellular ROS, *n* = 3. (**f**) The levels of IL-10, IL-6, IL-1β, and TNF-α were determined by biochemical assay kits, *n* = 6. Values are means ± SEM. Different letters and * represent significant differences (*p* < 0.05).

To identify the principal pathway mediating FMP protection, we employed ML385 (5 μM), a specific Nrf2 inhibitor. ML385 alone did not affect cell viability but markedly suppressed Nrf2 expression (Figure S3). In this set of experiments, the HFMP group (500 μM FMP), which previously demonstrated superior protective efficacy, was compared with the combined treatment of HFMP and the Nrf2 inhibitor ML385, while all other procedures remained unchanged (Figure 3a). The groups were designated as: Con (control), Mod (model, H_2_O_2_ alone), HFMP (500 μM FMP pretreatment), and IFMP (500 μM FMP pretreatment plus ML385 treatment). Notably, the FMP-induced increase in survival was completely abrogated by co-treatment with ML385 (Figure 3b). Although ML385 did not significantly alter HO-1 or NQO1 protein levels (Figure 3c), it substantially blunted FMP-enhanced antioxidant enzyme activities (T-AOC, CAT, GSH-Px, SOD) and elevated MDA content (Figure 3d). Likewise, the capacity of HFMP to reduce intracellular ROS and to modulate inflammatory mediators (upregulating IL-10 and downregulating IL-6 and IL-1β) was abolished by ML385 (Figure 3e,f), confirming that FMP counteracts H_2_O_2_-induced oxidative insult primarily via the Nrf2 signalling axis.

**Figure 4:**
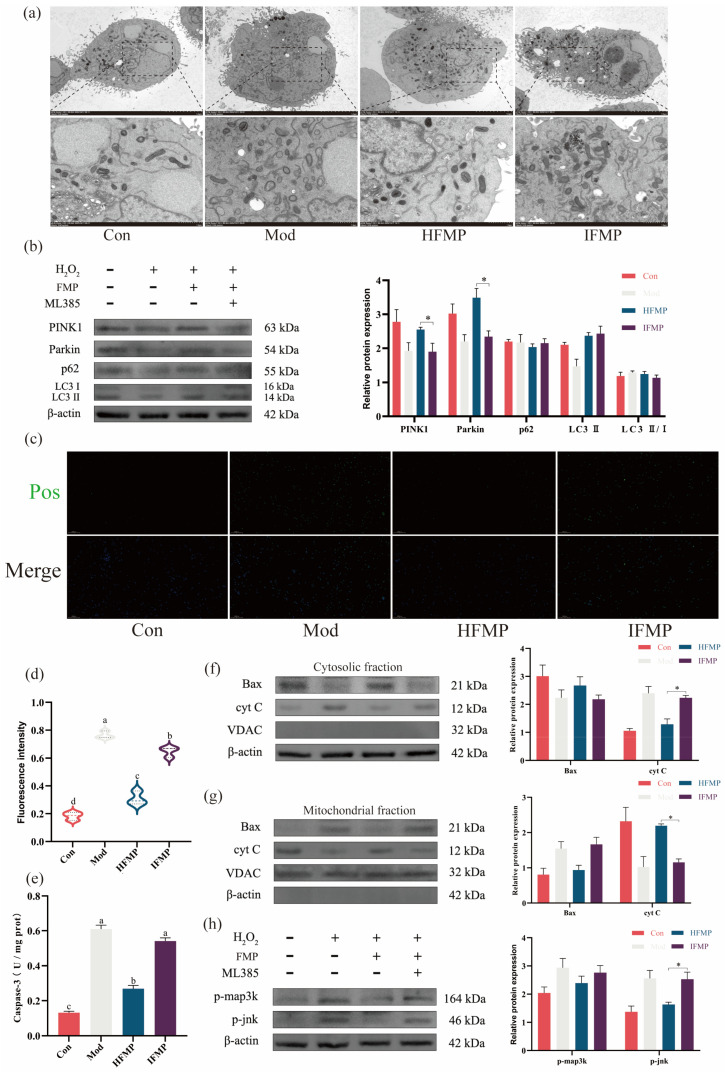
FMP alleviates H_2_O_2_-induced oxidative injury in IPEC-J2 cells via targeted activation of the Nrf2 pathway and concomitant modulation of mitophagy. (**a**) Cellular ultrastructure was visualized using TEM (2000 × magnification). (**b**) Western blotting determined the protein expression and quantitation of PINK1, Parkin, p62, LC3 I and LC3 II, *n* = 3. (**c**) Staining of apoptotic cells was determined by Tunel Assay Kit. (**d**) Relative content of apoptotic cells, *n* = 3. (**e**) The content of Cysteine-aspartic protease-3 was determined by Caspase-3 Assay Kit, *n* = 6. (**f**) Western blotting determined the protein expression and quantification of Bax and cyt C in the cytoplasm, *n* = 3. (**g**) Western blotting determined the protein expression and quantification of Bax and cyt C in the mitochondria, *n* = 3. (**h**) Western blotting determined the protein expression and quantification of p-MAP3K and p-JNK, *n* = 3. Values are means ± SEM. Different letters and * represent significant differences (*p* < 0.05).

We next determined whether FMP acts directly on mitophagy. The restoration of mitochondrial ultrastructure and upregulation of PINK1 and Parkin by HFMP were largely reversed by ML385 (Figure 4a,b), indicating that mitophagy is governed by Nrf2 rather than serving as a direct target of FMP. Consistent with this, ML385 reversed HFMP’s anti-apoptotic effects: it increased TUNEL-positive cells, elevated cytosolic cytochrome c while decreasing mitochondrial cytochrome c, suppressed phospho-JNK, and restored cleaved caspase-3. (Figure 4c–h). Collectively, these data demonstrate that FMP activates the Nrf2 pathway, which secondarily promotes mitophagy and restrains apoptosis to protect IPEC-J2 cells from oxidative injury.

### 3.4. FMP Administration Enhances Antioxidant Capacity and Intestinal Barrier Function in Piglets

**Figure 5:**
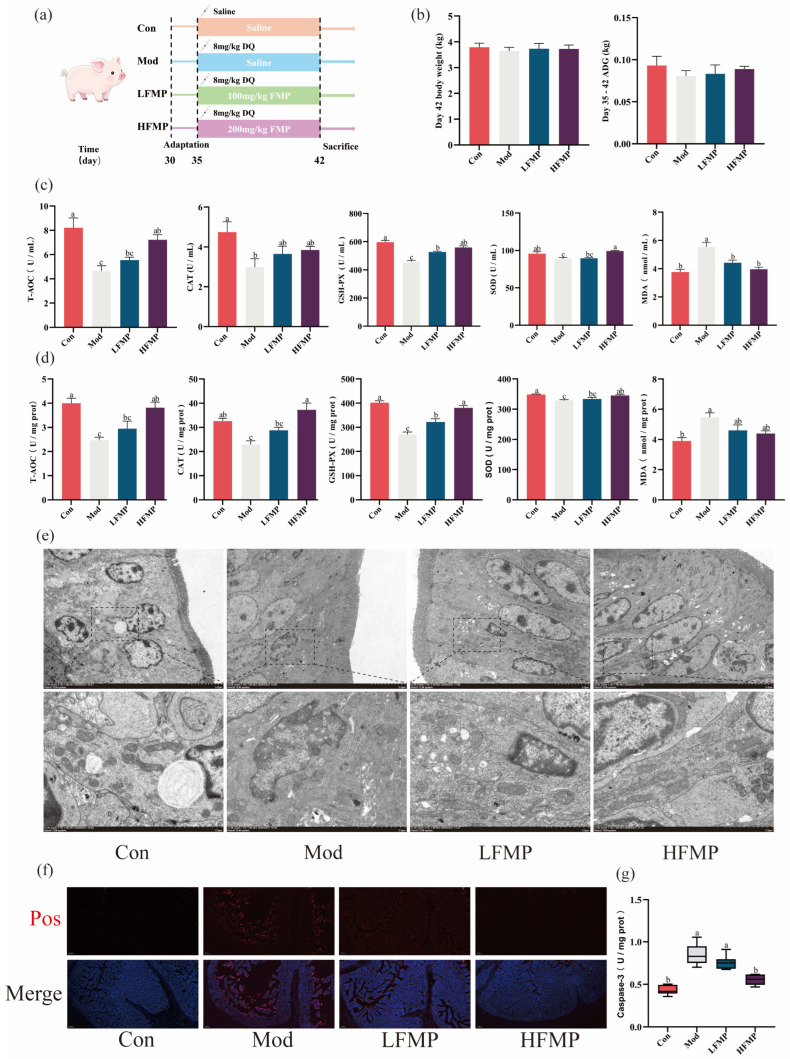
FMP administration enhances antioxidant capacity and intestinal barrier function in piglets. (**a**) The schematic diagram illustrates drug administration and experimental design in piglets. (**b**) Growth performance of piglets. ADG, average daily gain. *n* = 6. (**c**) The level of T-AOC, CAT, GSH-Px, SOD, and MDA in serum of piglets was determined by biochemical assay kits, *n* = 6. (**d**) The level of T-AOC, CAT, GSH-Px, SOD, and MDA in the jejunal mucosa of piglets was determined by biochemical assay kits, *n* = 6. (**e**) Representative pictures of TEM (2000 and 4000 × magnification) of jejunal tissue. (**f**) Staining and quantification of apoptotic cells in the jejunal mucosa of piglets, *n* = 3. (**g**) The content of Cysteine-aspartic protease-3 was determined by Caspase-3 Assay Kit, *n* = 6. Values are means ± SEM. Different letters represent significant differences (*p* < 0.05).

A diquat (DQ)-induced oxidative stress model was established in piglets (Con, Mod, LFMP, HFMP) to evaluate the in vivo protective efficacy of FMP (Figure 5a). DQ was administered intraperitoneally at a dose of 8 mg/kg body weight [19]. DQ challenge markedly decreased serum T-AOC, SOD, GSH-Px, and CAT activities and increased MDA, confirming systemic oxidative stress. HFMP significantly increased serum T-AOC and SOD levels, while both LFMP and HFMP significantly boosted GSH-Px and reduced MDA (*p* < 0.05), with parallel protective changes observed in jejunal tissue, but FMP did not significantly reduce MDA, while HFMP noticeably increased CAT (Figure 5c,d). Body weight and ADG were numerically higher in FMP-treated groups but did not reach statistical significance (Figure 5b). Ultrastructurally, FMP prevented DQ-induced severe mitochondrial swelling and disruption in jejunal epithelial cells (Figure 5e), and it suppressed apoptosis, as shown by reduced TUNEL-positive cells and caspase-3 cleavage (Figure 5f,g).

**Figure 6:**
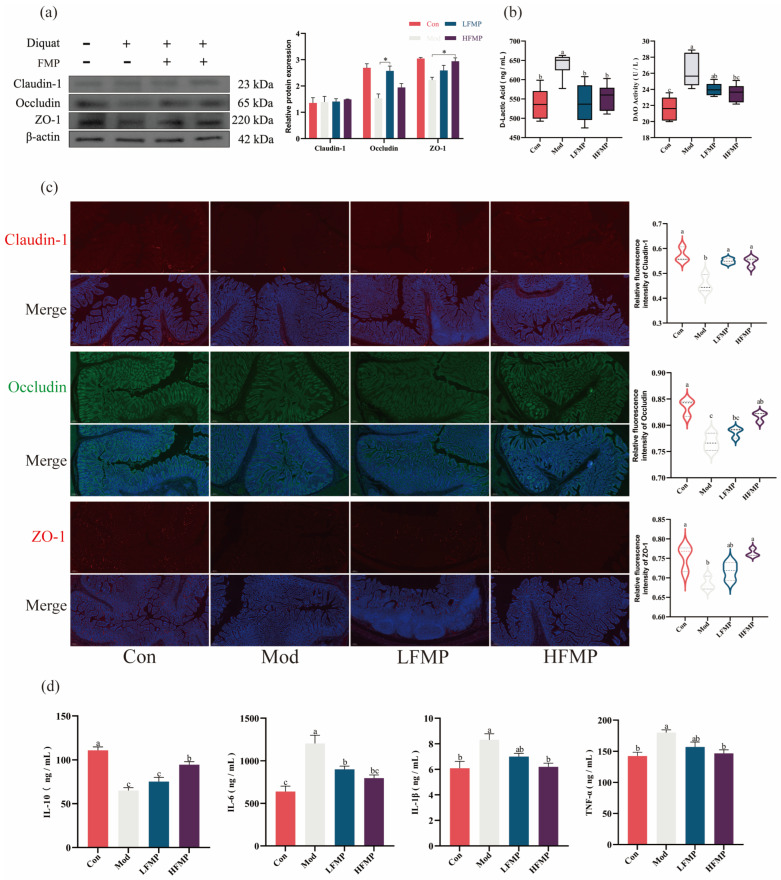
FMP enhances the intestinal barrier function of piglets. (**a**) Tight junction proteins (ZO-1, Occludin, and Claudin-1) were measured by Western blotting in the intestinal mucosa, *n* = 3. (**b**) Measurement of intestinal barrier indicators D-lactic acid and DAO in serum using biochemical assay kits, *n* = 6. (**c**) Representative immunofluorescence images of Claudin-1, Occludin, and ZO-1 and their quantification, *n* = 3. (**d**) The levels of IL-10, IL-6, IL-1β, and TNF-α in serum were measured using biochemical assay kits, *n* = 6. Values are means ± SEM. Different letters and * represent significant differences (*p* < 0.05).

FMP significantly upregulated jejunal tight junction proteins Occludin and ZO-1 (*p* < 0.05) and enhanced their immunofluorescence signals, indicating barrier reinforcement (Figure 6a,c). Consistently, FMP attenuated the DQ-evoked increases in serum D-lactate and DAO, markers of intestinal permeability (Figure 6b). Moreover, HFMP significantly elevated the anti-inflammatory cytokine IL-10 and reduced pro-inflammatory IL-6, IL-1β, and TNF-α (*p* < 0.05). Collectively, FMP enhances systemic and intestinal antioxidant capacity and effectively alleviates DQ-induced intestinal barrier injury in piglets.

### 3.5. Effect of FMP Treatment on the Gut Microbiota of Piglets Subjected to DQ-Induced Oxidative Stress

**Figure 7:**
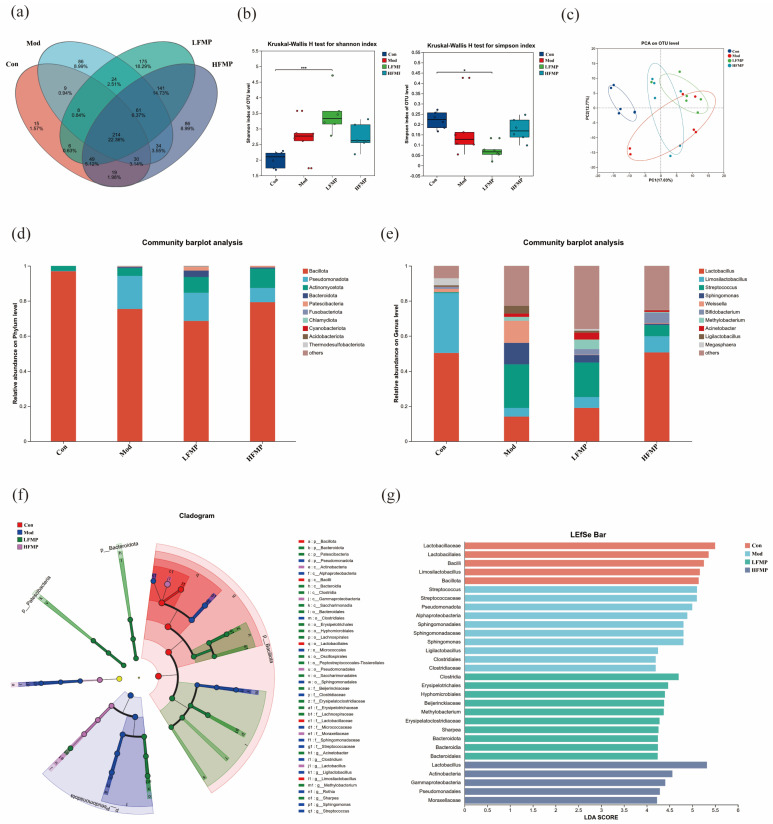
FMP Treatment on the Gut Microbiota of Piglets Subjected to DQ-Induced Oxidative Stress. (**a**) Venn diagram of OTU distribution, *n* = 6. (**b**) The alpha diversity indices (Shannon and Simpson) of intestinal microbiota, *n* = 6. (**c**) The beta diversity using the unweighted Unifrac Principal coordinates analysis (PCoA), *n* = 6. (**d**) Microbiota composition at the phylum level. (**e**) Microbiota composition at the genus level, *n* = 6. (**f**) Cladogram and (**g**) LDA distribution, *n* = 6. Values are means ± SEM. * represent significant differences (*p* < 0.05) and *** represents extremely significant difference (*p* < 0.001).

We examined the jejunal microbial composition. DQ challenge altered the overall gut microbiota structure, as reflected by OTU Venn diagrams, and alpha and beta diversity metrics, while FMP treatment groups (LFMP and HFMP) showed a partial restoration trajectory relative to the model group, but these changes are not that noticeable (Figure 7a–c). At the phylum level, DQ reduced the relative abundance of Firmicutes and increased Proteobacteria; FMP partially restored Firmicutes dominance and elevated Actinobacteria (Figure 7d). DQ diminished the genus Lactobacillus, whereas, in the HFMP group, it reestablished its predominance (Figure 7e). LEfSe analysis (LDA > 4.0) revealed that the control group was characterized by Lactobacillus-affiliated lineages and the model group by an expansion of Streptococcus and Proteobacteria; in the LFMP group, Clostridia were abundant; in the HFMP group, Lactobacillus was somewhat restored. Overall, FMP improved the gut microbiota imbalance caused by DQ to some extent by regulating specific microbial taxa.

## 4. Discussion

In intensive swine production, weaned piglets are subjected to stressors that predispose them to intestinal oxidative injury, leading to diarrhea, growth retardation, and economic losses [20,21]. Insect-derived bioactive peptides, which offer high feed efficiency and multifaceted bioactivities, have emerged as promising green antioxidant feed additives [22]. However, the molecular targets and regulatory pathways of maggot-derived antioxidant peptides remain largely unelucidated, hindering their application. Here, we employed a novel maggot-derived antioxidant peptide, FMP (amino acid sequence YDCFQQN), and systematically elucidated its protective mechanisms in IPEC-J2 cells and a weaned piglet model. Our findings delineate a multi-tiered mechanism whereby FMP activates the Nrf2 signaling pathway, enhances mitophagy, restores barrier function, and modulates the gut microbiota. The Nrf2/Keap1 pathway is the central defense against oxidative stress. Under basal conditions, Keap1 sequesters Nrf2 and promotes its proteasomal degradation; upon oxidative challenge, Nrf2 dissociates, translocates to the nucleus, and drives transcription of cytoprotective enzymes such as HO-1 and NQO1 [23,24]. In livestock, early weaning suppresses the Keap1/Nrf2 pathway, whereas dietary Zn-L-selenomethionine activates it [25,26]. In IPEC-J2 cells, FMP pretreatment significantly counteracted H_2_O_2_-induced cytotoxicity and ROS accumulation, elevated T-AOC, CAT, GSH-Px, and SOD activities, reduced MDA levels, and upregulated Nrf2, HO-1, and NQO1 while downregulating Keap1. Critically, the Nrf2 inhibitor ML385 virtually abrogated these protective effects, confirming that Nrf2 activation is the core mechanism of FMP action [27]. Inhibition of Nrf2 did not significantly alter HO-1 or NQO1 protein levels, which may be attributed to post-transcriptional regulation or other compensatory mechanisms that warrant further investigation.

Mitochondria are both the primary source and target of ROS. Excessive ROS damages mitochondrial components, initiating a vicious cycle that amplifies oxidative stress [28,29]. Mitophagy clears damaged mitochondria via the PINK1/Parkin pathway, preserving cellular homeostasis [11,30,31]. Nrf2 and mitophagy exhibit bidirectional regulation, with Nrf2 directly enhancing PINK1 transcription [8,32]. In our study, H_2_O_2_ induced profound mitochondrial damage in IPEC-J2 cells, whereas FMP preserved mitochondrial ultrastructure and increased PINK1, Parkin, and LC3-II expression. ML385 reversed these benefits, establishing that FMP-enhanced mitophagy requires Nrf2 activation. Mitochondrial dysfunction triggers apoptosis through cyt C release and apoptosome assembly [33,34]. FMP suppressed H_2_O_2_-induced cyt C release, Bax mitochondrial translocation, p-JNK upregulation, and caspase-3 cleavage; ML385 co-treatment abolished these anti-apoptotic effects. Thus, FMP-mediated inhibition of apoptosis is a downstream consequence of Nrf2-dependent mitophagy and mitochondrial homeostasis.

The intestinal epithelial barrier depends on tight junction proteins such as Occludin and ZO-1, and oxidative stress disrupts barrier integrity via multiple mechanisms, promoting inflammation and growth suppression [35,36,37]. In weaned piglets, diquat (DQ) challenge markedly reduced serum T-AOC, SOD, GSH-Px, and CAT activities, elevated MDA, induced jejunal mitochondrial injury, and increased intestinal permeability markers D-lactic acid and DAO. Oral FMP significantly restored antioxidant enzyme activities, reduced MDA, preserved mitochondrial morphology, and rescued Occludin and ZO-1 expression. FMP also decreased serum IL-6, IL-1β, and TNF-α while increasing IL-10, consistent with reported taurine effects [38]. These results demonstrate that FMP protects against DQ-induced intestinal injury by enhancing systemic antioxidant defenses, maintaining barrier integrity, and attenuating inflammation. While FMP-treated animals exhibited numerically higher final body weights and average daily gains, these differences were not statistically significant, possibly due to the limited experimental duration or sample size; extended studies are required to evaluate growth-promoting potential.

Both weaning stress and oxidative challenge disrupt the gut microbiota, reducing beneficial taxa and enriching pathogens, which exacerbates barrier dysfunction [39,40]. The intestinal oxidative stress caused by DQ can further disrupt the gut microenvironment, leading to changes in the composition and diversity of the gut microbiota. In our study, DQ challenge decreased the relative abundance of Firmicutes and Lactobacillus while increasing Proteobacteria and Streptococcus in the jejunal microbiota. As a core beneficial genus, reduced Lactobacillus signifies microecological disturbance [41]. FMP intervention, particularly at the high dose, partially restored Lactobacillus dominance and increased Actinobacteria abundance. LEfSe analysis confirmed that FMP-treated groups diverged from the model group, indicating partial mitigation of DQ-induced dysbiosis. Whether FMP alters microbial composition through direct antimicrobial/prebiotic activity or indirectly by improving the intestinal redox environment requires further study. Additionally, the present analysis was limited to jejunal digesta; future work should encompass other intestinal compartments and employ metagenomic approaches for a comprehensive assessment.

Synthetic antioxidants, notably butylated hydroxytoluene (BHT) and butylated hydroxyanisole (BHA), encounter progressively rigorous regulatory constraints in feed formulations, a consequence largely ascribed to their documented toxicological risks [14,42]. Against this backdrop, bioactive peptides of natural origin have garnered considerable research attention as promising alternative antioxidant candidates, owing to their favourable safety characteristics and potent biological activities. In the present study, we systematically elucidated for the first time the core mechanism framework by which fly maggot-derived antioxidant peptide (FMP) exerts its protective effects. Specifically, FMP was demonstrated to engage the Keap1/Nrf2 signaling axis, which subsequently facilitates the coordinated upregulation of PINK1/Parkin-directed mitophagy, thereby safeguarding mitochondrial integrity. This signaling cascade culminates in the inhibition of the mitochondrial apoptotic machinery and inflammatory signaling, the reestablishment of intestinal tight junction barrier function, and the remodelling of gut microbial community composition. In weaned piglets, FMP significantly enhanced antioxidant capacity and improved intestinal health, thereby exhibiting tangible practical utility and presenting a novel strategy for promoting green and health-oriented piglet husbandry. In conclusion, this study not only enriches the theoretical framework governing the regulation of intestinal health in livestock and poultry by insect-derived antioxidant peptides but also identifies a promising candidate for a novel green feed additive aimed at ameliorating oxidative stress in weaned piglets.

## 5. Conclusions

**Figure 8:**
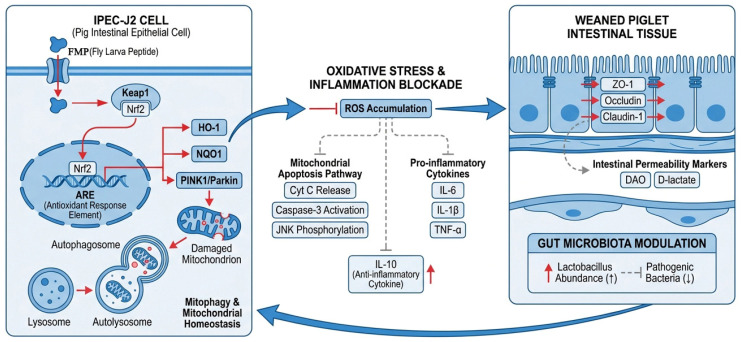
Mechanism: FMP alleviates intestinal oxidative injury in weaned piglets.

This study demonstrates that the fly maggot-derived antioxidant peptide FMP (amino acid sequence: YDCFQQN) effectively attenuates H_2_O_2_-induced oxidative injury in IPEC-J2 cells and alleviates diquat-induced intestinal oxidative stress in weaned piglets. The core underlying mechanism involves the activation of the Keap1/Nrf2 signaling pathway by FMP, which synergistically upregulates PINK1/Parkin-mediated mitophagy to preserve mitochondrial homeostasis (Figure 8). This cascade subsequently suppresses the mitochondrial apoptotic pathway and inflammatory responses, restores intestinal tight junction barrier integrity, and ameliorates the compositional structure of the gut microbiota. These findings provide a theoretical foundation for the application of maggot-derived antioxidant peptides as green feed additives aimed at mitigating oxidative stress in piglets.

## Data Availability

Raw sequencing data are available in the NCBI SRA database under accession number PRJNA1463741; further inquiries can be directed to the corresponding authors.

## Supplementary Materials

The following supporting information can be downloaded at https://www.mdpi.com/article/10.3390/antiox15070791/s1, Figure S1: Establishment of oxidative stress model in IPEC-J2 cells; Figure S2: Pre-protective effect of FMP on H_2_O_2_-induced damage in IPEC-J2 cells; Figure S3: The effect of the inhibitor ML385 on Nrf2 protein levels in IPEC-J2 cells.

## Abbreviations

The following abbreviations are used in this manuscript:

FMP	Fly maggot-derived antioxidant peptide
NAC	N-Acetylcysteine
DQ	Diquat
BW	Body weight
ADG	Average daily gain
T-AOC	Total antioxidant capacity
GSH-Px	Glutathione peroxidase
T-SOD	Total superoxide dismutase
MDA	Malondialdehyde
H&E	hematoxylin and eosin
OTUs	Operational taxonomic units
ANOVA	Analyzed by one-way analysis of variance
HSD	Honestly significant difference
PCoA	Principal coordinate analysis
PERMANOVA	Permutational multivariate analysis of variance
LEfSe	Linear discriminant analysis effect size
DAO	Diamine oxidase
ROS	Reactive oxygen species
ARE	Antioxidant response elements
