# Supplementary material for: The Fly Maggot Antioxidant Peptide (FMP) Alleviates Oxidative Damage in the Intestines of Weaned Piglets by Enhancing Mitochondrial Autophagy Through Activation of the Nrf2 Signaling Pathway
Source: Antioxidants (Basel). 2026 Jun 24;15(7):791. doi: 10.3390/antiox15070791 (PMC13404538; doi:10.3390/antiox15070791)
Supplement: Supplementary file 1 [file antioxidants-15-00791-s001.zip › antioxidants-4361866-supplementary.pdf]

The Fly Maggot Antioxidant Peptide (FMP) Alleviates Oxidative Damage in the Intestines of Weaned Piglets by Enhancing Mitochondrial Autophagy Through Activation of the Nrf2 Signaling Pathway

Xingke Wang, Ruiying Bao, Qingchao Yang, Qian Yang, Sheng Gao, Qingying Cai, Yang Zhang, Haiwen Zhang, Huiyu Shi and Xuemei Wang *

Animal Nutrition and Feed Laboratory, School of Tropical Agriculture and Forestry, Hainan University, Danzhou 571737, China; xkwang28@hainanu.edu.cn (X.W.); 23110710000036@hainanu.edu.cn (R.B.); 24220951330003@hainanu.edu.cn (Q.Y.); 20233004953@hainanu.edu.cn (Q.Y.); 23210905000009@hainanu.edu.cn (S.G.); 23220951330001@hainanu.edu.cn (Q.C.); 25210905000015@hainanu.edu.cn (Y.Z.); hwzhang@hainanu.edu.cn (H.Z.); 993978@hainanu.edu.cn (H.S.)
* Correspondence: wangxuemei@hainanu.edu.cn

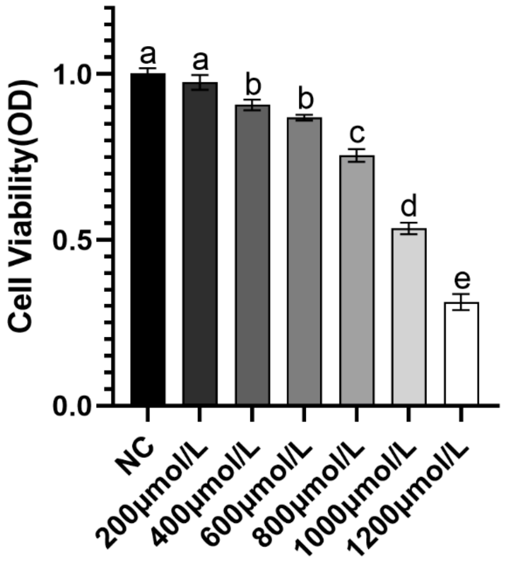

Figure S1. Establishment of oxidative stress model in IPEC-J2 cells. The effect of incubation duration and concentration of H_2O_2 on IPEC-J2 cell viability was determined by CCK-8 assay. Values are means ± SEM. Different letters represent significant differences ($p < 0.05$).

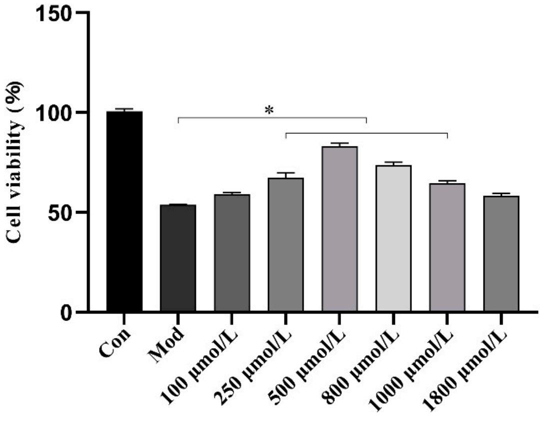

Figure S2. Pre-protective effect of fly maggot-derived antioxidant peptide (FMP) on H_2O_2-induced damage in IPEC-J2 cells. The effect of incubation duration and concentration of FTP on the viability

of IPEC-J2 cells pretreated with H_2O_2 was determined by CCK-8 assay. Values are means ± SEM. * represent significant differences ($p < 0.05$).

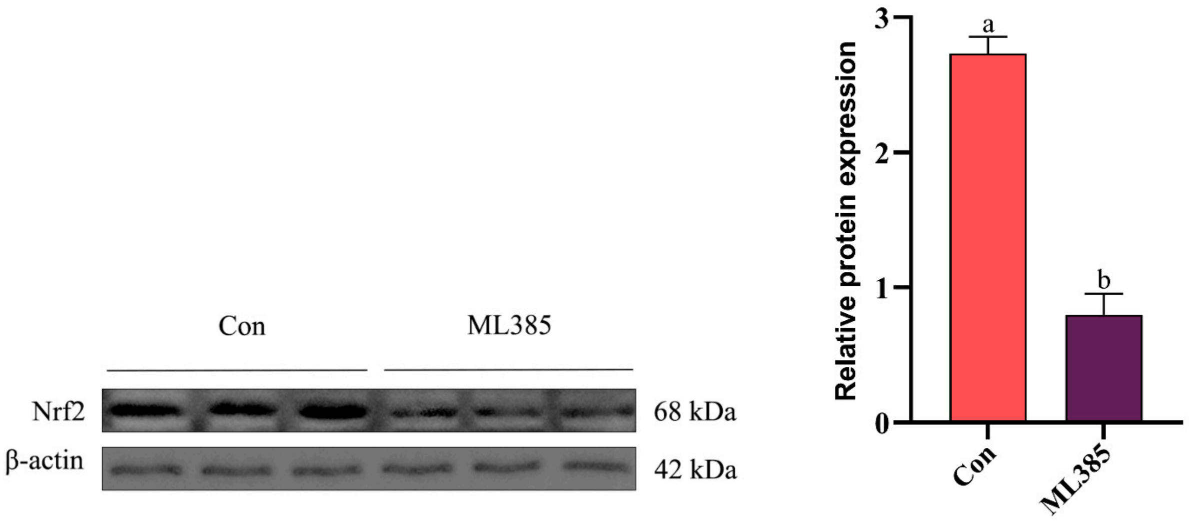

Figure S3. The effect of the inhibitor ML385 on Nrf2 protein levels in IPEC-J2 cells. Western blotting determined the protein expression and quantitation of Nrf2. Values are means ± SEM. Different letters represent significant differences ($p < 0.05$).
